# Miscellany

**Published:** 1891-08

**Authors:** 


### Miscellany.

MALTINE.—A striking feature of the new advertisement of this nutritive tonic, on cover page iv., is the design of a Maltese cross, in which the play on the word "Maltine" will be noted. It was under the banner of the Maltese Cross that the Crusaders of the middle ages vanquished the followers of the Crescent, and this form of cross was adopted by the hospitallers of the Order of the Knights of St. John of Jerusalem, who were among the first founders of institutions established to ameliorate the physical ills of mankind. After their occupation of the Island of Malta, which they held for centuries against the assaults of the Infidel, this symbol came to be known as the "Maltese Cross." It is emblematical of the success of merit, of practical benefit to mankind, and of ability to resist attack. The appropriateness of the use of this design in connection with a valuable or constructive nutrient tonic, is therefore apparent.

AN OPEN COMPETITIVE EXAMINATION of candidates for Junior Assistant Physician in any of the State Hospitals and Asylums, will be held at the office of the Civil Service Commission, Albany, N. Y., Thursday, August 20, 1891, commencing at ten o'clock A. M. A candidate for the position must be a citizen of the State of New York, at least 21 years of age, and have had at least one year's experience in a hospital, or three years' experience in the general practice of medicine. For application blanks, address the Secretary of the New York Civil Service Commission, Albany, N. Y.

JOHN B. RILEY,
ALBANY, N. Y., July 3, 1891. *Chief Examiner.*

